# An efficient computational chemistry approach to generating negative data for drug discovery pipeline validation

**DOI:** 10.3389/fbinf.2026.1756279

**Published:** 2026-02-27

**Authors:** Stefan M. Ivanov

**Affiliations:** 1 Faculty of Pharmacy, Medical University of Sofia, Sofia, Bulgaria; 2 Centre of Excellence in Informatics and Information and Communication Technologies, Sofia, Bulgaria; 3 Institute of Biophysics and Biomedical Engineering, Bulgarian Academy of Sciences, Sofia, Bulgaria

**Keywords:** cheminformatics, gnina, MM-PBSA, molecular docking, molecular dynamics simulation, negative data, recovery plots, VHTS

## Abstract

Modern virtual high-throughput screening (VHTS) pipelines can be suboptimally validated, with no rigorous studies conclusively demonstrating that every one of their steps reliably adds increasing enrichment atop the baseline random hit rate. Moreover, what little benchmarking studies are available primarily focus on the docking aspect of the pipelines, which is usually only the beginning or near the beginning, and even there, authors tend to use flawed data sets that artificially inflate performance metrics. Herein, we present an alternative method to pipeline validation and data set generation that requires no additional experimental work and expenditure, yet offers vast amounts of negative data that can be used in VHTS pipeline validation. By randomizing ligands across published experimental structures and generating structural isomers of known binders, practically unlimited amounts of negative data can be generated. Such sets of positive and negative data points match closely in key molecular properties and are well suited to pipeline validation. Once such sets are generated, they are to be run through any proposed pipeline, assessing performance at every step. We stress the importance of using negative data of adequate quality and quantity in validation studies to definitively and verifiably demonstrate the utility of a given tool or workflow. Our goal is to help distinguish tools and pipelines that truly accelerate hit discovery and lead optimization from ones that promise to do so but actually do not, whereupon academia and industry can begin to tackle the many unaddressed medical needs of the 21st century.

## Introduction

The cost of developing new drugs ranges from hundreds of millions to billions of dollars (Sertkaya et al., 2024). Unsurprisingly, there is no shortage of conditions that lack treatment or an effective vaccine or require new and better drugs (Kundra et al., 2021; Sung et al., 2021; Qiu et al., 2009; Ivanov et al., 2020; Brooks, 2024; Draper et al., 2018; The Lancet Gastroenterology and Hepatology, 2021; Nasir et al., 2017). Preclinical development accounts for a significant share of the costs, time, and effort involved in modern drug development (Schlander et al., 2021). Indeed, it can take significant time, effort, and resources just to find a hit molecule for a particular target. The baseline random hit rates vary from protein to protein and from ligand set to ligand set, but most high-throughput screens report hit rates well below 1%. For example, a compilation of 21 screening decks (Tran-Nguyen et al., 2020) deposited in the PubChem BioAssay database (Wang et al., 2017) shows a random hit rate as low as 0.018% against the κ_1_-opioid receptor–out of 284,220 compounds tested, only 51 proved active (assay ID 1777). Moreover, experimental screens where no actives have been found, i.e., have a hit rate of 0, are likely to not be reported at all. While hit rates around or even above 1% have been published (Tran-Nguyen et al., 2020), for any given protein target and commercially available set of small molecules, it would be reasonable to expect a random hit rate of 1% or lower, more likely 0.1% or lower, if and until sufficient evidence becomes available to suggest otherwise. Indeed, if random hit rates around 1% were typical, hit discovery would likely have been easy–for every 1000 random ligands tested, one would obtain about 10 different hits to kickstart a drug development program. Experimentalists have learned from experience that this many hits often require testing tens if not hundreds of thousands of molecules against the target of interest.

In an effort to streamline hit discovery and reduce costs, many practitioners have turned to *in silico* modeling and simulation which has given rise to computer-aided drug design (CADD (Niazi and Mariam, 2024)) and computer-aided molecular design (CAMD (Austin et al., 2016)). In a hit discovery context, structure-based virtual high-throughput screening (VHTS) pipelines (Carlsson and Luttens, 2024; Lionta et al., 2014) typically employ a funnel-shaped model where the search begins with a large number of small molecules, often commercially available collections, which may be filtered for pan-assay interference compounds (PAINS (Baell and Holloway, 2010)) and undesirable properties such as molecular volume or weight that are too large or too small for the binding site of the target (usually a protein molecule or a complex of proteins). The remaining ligands are then initially subjected to a crude and inexpensive computational technique to try to filter out the majority of the likely nonbinders, typically molecular docking (Zhang et al., 2022). Subsequent steps in the pipeline employ increasingly sophisticated, computationally costly, and theoretically rigorous techniques on a progressively decreasing pool of molecules, with the intermediate steps often involving end-state free energy techniques such as the linear interaction energy (LIE) method (Su et al., 2007; Brandsdal et al., 2003), molecular mechanics–generalized Born (MM-GBSA) or molecular mechanics–Poisson-Boltzmann (MM-PBSA) calculations (Genheden and Ryde, 2015; Maffucci and Contini, 2013; Maraf et al., 2024). The final stages of the pipeline may involve theoretically rigorous absolute binding free energy techniques (ABFEs (Aldeghi et al., 2018)) on one to several hundred molecules, the most promising of which are then purchased for experimental testing and verification.

While molecular docking (Paggi et al., 2024) and dynamics (Bowers et al., 2006) have produced new knowledge (Ivanov et al., 2016; Ivanov et al., 2019a; Dodd et al., 2020; Yu et al., 2023), insights (Ivanov et al., 2020; Ivanov et al., 2019b; Bhakat and Söderhjelm, 2017; Kumalo et al., 2016; Childers et al., 2017; Atanasova et al., 2024; Marković et al., 2024; Spassov et al., 2024; Spassov, 2024; Atanasova, 2025; Philipova et al., 2025), and hits (Atanasova et al., 2022; Stavrakov et al., 2020), much remains to be desired. A thorough benchmarking analysis estimating the enrichment provided by these techniques at every step of a VHTS pipeline is not available. Benchmarking studies have been published primarily focusing on docking (Bertrand and Triballeau, 2005). Yet, even there, much of the results remain suspect due to biases in the composition of the positive and negative data. For example, it has been pointed out that in the directory of useful decoys data set (DUD (Huang et al., 2006)), actives spread over a few dominant scaffolds (analog bias (Good and Oprea, 2008)) and that decoys exhibit net charges different from active molecules (Hawkins et al., 2008). Subsequent efforts have produced the directory of useful decoys data set-enhanced (DUD-E (Mysinger et al., 2012)), DUDE-Z (Stein et al., 2021), and maximum unbiased validation (MUV (Rohrer and Baumann, 2009)) sets which, nevertheless, suffer significant biases (Wallach and Heifets, 2018). For DUD-E, differences in key molecular properties such as polar surface area and hydrogen bond donor counts between actives and nonactives have been reported (Chaput et al., 2016). Moreover, the actives in DUD-E are biased toward higher potency than what is observed during prospective high-throughput screens (Tran-Nguyen et al., 2020). Analyses on commonly used data sets reveal that artificially inflated enrichments are often the result of analogue bias (Xia et al., 2015) or 2D bias (Vázquez et al., 2024). Imbalances in properties between actives and decoys deceptively inflate enrichment numbers and other performance metrics in benchmarking studies using these sets and overestimate the utility of the tools being assessed. This has been known for some time now and practitioners have become weary of overly optimistic reports (Wallach et al., 2015; Pereira et al., 2016), knowing that performance in a truly prospective screen is likely to be much lower than what is reported in the literature. This has provided the impetus for setting up the Critical Assessment of Computational Hit-finding Experiments (CACHE) challenges (Ackloo et al., 2022) where computational chemists are tasked with finding novel hits for targets of interest and optimizing them in a truly prospective setting. This produces an objective assessment of their methods’ capabilities unlike *post hoc* reports where authors may omit or gloss over certain failings and exaggerate performance. We explicitly state that any deficiencies we point out here are not a criticism of the authors we cite who have done important foundational work in the field of VHTS. Rather, these serve as an illustration of the inherent difficulty of the problem of finely matching decoys to actives in a highly-dimensional property hyperspace.

So far, the standard approach to data set generation has been to experimentally measure different ligands against targets of interest. This is an expensive approach that inevitably results in differences between actives and inactives (and hence biases), as it is not possible to perfectly match binders to nonbinders in the vastness of chemical property space. Indeed, while authors may be somewhat successful in obtaining decoys with similar molecular weight or partition coefficient (logP) distributions to those in their active sets, it quickly becomes nearly impossible to obtain a good match when one also begins to consider other molecular properties such as number of rotatable bonds (henceforth also referred to as rotors), polar and nonpolar molecular surface area, net charge, number of hydrogen bond donors and acceptors, etc. Moreover, one of the key molecular properties–the number of rotatable bonds a ligand has–is a rather coarse metric. One could easily imagine a scenario where two molecules have the same number of rotatable bonds but which differ greatly in their flexibility (Wicker and Cooper, 2016), for example, two small aromatic rings being separated by a given number of rotatable bonds versus two large aromatic systems being separated by the same number of rotors. Moreover, thus far no one has described a means to thoroughly measure and validate the performance of and value added by every step in a VHTS pipeline. In this work, we describe an efficient approach to producing negative data for VHTS validation. We also illustrate a means of evaluating performance at every step of the pipeline, which makes possible comparing different techniques and optimizing VHTS pipelines by swapping out less efficient techniques in favor of more productive ones. Moreover, our method allows validating modifications to VHTS pipelines as well as quantitatively comparing the performance of different pipelines.

Our approach is two-fold. First, rather than measuring ligand activities against a target of interest, we take actives from PDBbind (Wang R. et al., 2004) structures. PDBbind is the largest curated set of protein–ligand structures and affinities compiled from literature. To generate a corresponding negative set, we randomize ligands such that every ligand is paired with a noncognate protein structure. This is the only way to achieve a perfect match in chemical properties between positives and negatives, as the positive and negative ligand set contain the exact same molecules. Moreover, this approach has the added benefit that not only does every ligand serve as its own control, but so does every protein.

Second, given a protein–ligand crystal or NMR structure, we generate negative data by producing constitutional isomers (also referred to in chemistry as structural isomers) of the binder with MAYGEN (Yirik et al., 2021). Because actives and decoys have the same heavy atom content, they are much closer in key properties than any set that is obtained by experimentally testing for random actives and inactives which would in all likelihood be completely unrelated. Using the standard approach to data set construction, actives and decoys would likely have very different weights, numbers of rotors, polar and nonpolar molecular surfaces, or exhibit significant differences in some other key property. By generating structural isomers of the binder, many of these imbalances, such as molecular weight or molecular surfaces, are either greatly ameliorated or avoided completely (Ivanov, 2026). Ivanov (2026) explores property matching between actives, experimental decoys, and isomers at much greater depth; it also features example code to filter ligands for or against desired/undesired properties or property ranges.

## Methods

### PDBbind-derived set

We selected 29 monomeric structures with available dissociation constant (*K*_D_) values from the refined set of PDBbind 2019, thus ensuring that we are working with high quality, reliable structures. For every protein–ligand structure, we generated 25 more structures by docking different noncognate ligands into the binding site of the protein with Gnina 1.0 (McNutt et al., 2021), using the cognate ligand as a reference to define the binding site. Typically, a random ligand can be safely assumed to be a nonbinder (decoy). However, some of the PDB structures in our set were degenerate in that they contained the same protein (as evidenced by the Uniprot ID (Apweiler et al., 2004)). Therefore, we searched our set for instances where a ligand is docked to different structures of the same protein it is known to bind from the PDBbind structure it originally came from. This resulted in a final set of 29 cognate, binding protein–ligand pairs (the crystal structures), as well as 26 binding pairs from the randomizations. Out of the 725 randomized pairs, we have 26 binding and 699 nonbinding (decoy) pairs. All ligands were docked to the proteins from the 29 PDBbind structures. In 29 cases, this meant ligands were docked to the protein from the crystal structure they came from (redocking); in 26 cases, this meant docking to a different protein conformation (crossdocking). As an example, the ligand from the 5NK6 crystal structure (Heinzlmeir et al., 2017) was docked to the same protein–ephrin type-A receptor 2 – but from a different crystal (5IA3 (Heinzlmeir et al., 2016)) into the binding site defined by the ligand in 5IA3. All protein–ligand pairs and their binding status (binding/nonbinding) are given in Supplementary Table 1.

All 754 docking runs (29 redockings and 725 crossdockings) were performed with Gnina, using 8 Monte Carlo chains during sampling and adding 4 Å of buffer space to the auto-generated box defined by the cognate reference ligand, and expanding the box by an additional 1 Å if needed to make sure the input ligand conformation can freely rotate within the box. Gnina provides 3 scores for every pose it outputs–the predicted affinity (in kcal/mol), a convolutional neural network (CNN) pose score–a neural network derived probability that the pose has a low root mean square deviation (RMSD) to the true binding pose–and a CNN affinity–a neural network estimate of the affinity, given in pK units. For the 754 structures originating from PDBbind (29 redockings and 725 crossdockings), we took the highest-scoring ligand pose for every protein–ligand pair using the CNN pose score and subjected the complexes to molecular dynamics (MD) simulations and MM-GBSA and MM-PBSA calculations.

### MAYGEN set

For the MAYGEN decoy generation, we began by taking the ligands from the 4QSW (Chaikuad et al., 2014), 5A5N (Demont et al., 2015), and 6EPU (Dolbois et al., 2020) crystal structures which all bind the same protein - ATPase family AAA domain-containing protein 2 (ATAD2). For every ligand, we generated 25 structural isomer decoys using MAYGEN (see Supplementary Figure S1 and Supplementary Table 1). For this work, we chose MAYGEN because it is free and open source; any other proprietary or nonproprietary tool for generating constitutional isomers (Steinbeck, 2001; Badertscher et al., 2000; Nuzil et al., 1991; Gugisch et al., 2015) could be used. The ligands were converted from SMILES to sdf format and protonated corresponding to neutral pH with Open Babel (O’Boyle et al., 2011). SMILES strings for all MAYGEN ligands are given in Supplementary Table 1. Finally, all 3 binders and 75 nonbinders were crossdocked to ATAD2 using the 4TU4 (Poncet-Montange et al., 2015) crystal structure and the binding site defined by the 4TU4 ligand which also binds the same site as the other three binders. Docking was performed with Gnina using identical settings as was done with the PDBbind set. However, here, for every ligand we took the top-scoring pose according to Gnina affinity, CNN pose score, and CNN affinity. These were then subjected to molecular dynamics and MM-PB(GB)SA analysis. Gnina is a stochastic docking tool which means that different runs produce different predictions for the binding pose. Therefore, for both the PDBbind and MAYGEN set, we performed molecular docking in 4 independent replicas followed by molecular dynamics on the best (i.e., highest) scoring poses from every replica. For the PDBbind set, we performed 4 MD replicas starting from the highest scoring conformations by CNN pose score from the 4 docking runs; for the MAYGEN set, we performed 4 MD runs on the best scoring poses using all three Gnina descriptors or 12 MD runs in total for every ligand.

### Molecular dynamics and MM-PB(GB)SA calculations

Ligand structures were processed with *antechamber* and *parmchk2* from Amber18 (Case et al., 2005) to generate Amber-compatible mol2 files containing AM1-BCC charges (Jakalian et al., 2000) and GAFF2.11 atom types (Wang JM. et al., 2004). Proteins were parameterized with the ff14SB force field (Maier et al., 2015) using *tleap*. Protein–ligand complexes were solvated with TIP3P water (Jorgensen et al., 1983) in a truncated octahedral box with a wall distance of 13 Å from the outermost atoms; NaCl (Joung and Cheatham, 2008) was added to neutralize the system net charge and reach a concentration of 150 mmol. The structures were then subjected to 50,000 steps of energy minimization with restraints of 3 kcal*mol^−1^ *Å^-2^ on protein heavy atoms, followed by constant volume heating to 300 K over a period of 1 ns, a density equilibration phase of 1 ns, followed by 5 ns of equilibration at constant pressure and temperature (NPT conditions), maintained with the Berendsen barostat (Berendsen et al., 1984) and the Langevin thermostat, and a production phase of 25 ns of NPT simulation. Collision frequencies and pressure relaxation times were set as in previous work (Ivanov et al., 2019a). As before, a cutoff of 12 Å was used for both van der Waals and electrostatic interactions; long-range electrostatics beyond the real-space cutoff were calculated with the particle-mesh Ewald (PME (Darden et al., 1993)) scheme, and bonds to hydrogen were constrained with SHAKE (Ciccotti and Ryckaert, 1986), allowing for a 2 fs time step. All systems were simulated with heating, density equilibration, preproduction dynamics, and production dynamics under periodic boundary conditions; only the minimization stage was run under nonperiodic conditions. During production dynamics, frames were saved every 100 ps for a total of 250 frames per trajectory.

We then performed MM-GBSA and MM-PBSA calculations on the 250 protein–ligand frames from the production stage of every trajectory to compute the enthalpy of binding (ΔH) between the proteins and ligands. Full details about these calculations are given in our previous publication on RNase A–ligand binding (Ivanov et al., 2019a). Finally, actives and decoys were ranked by their docking and MM-PB(GB)SA scores. These were compared to assess their performance relative to each other.

## Results

### PDBbind-derived set

**FIGURE 1:**
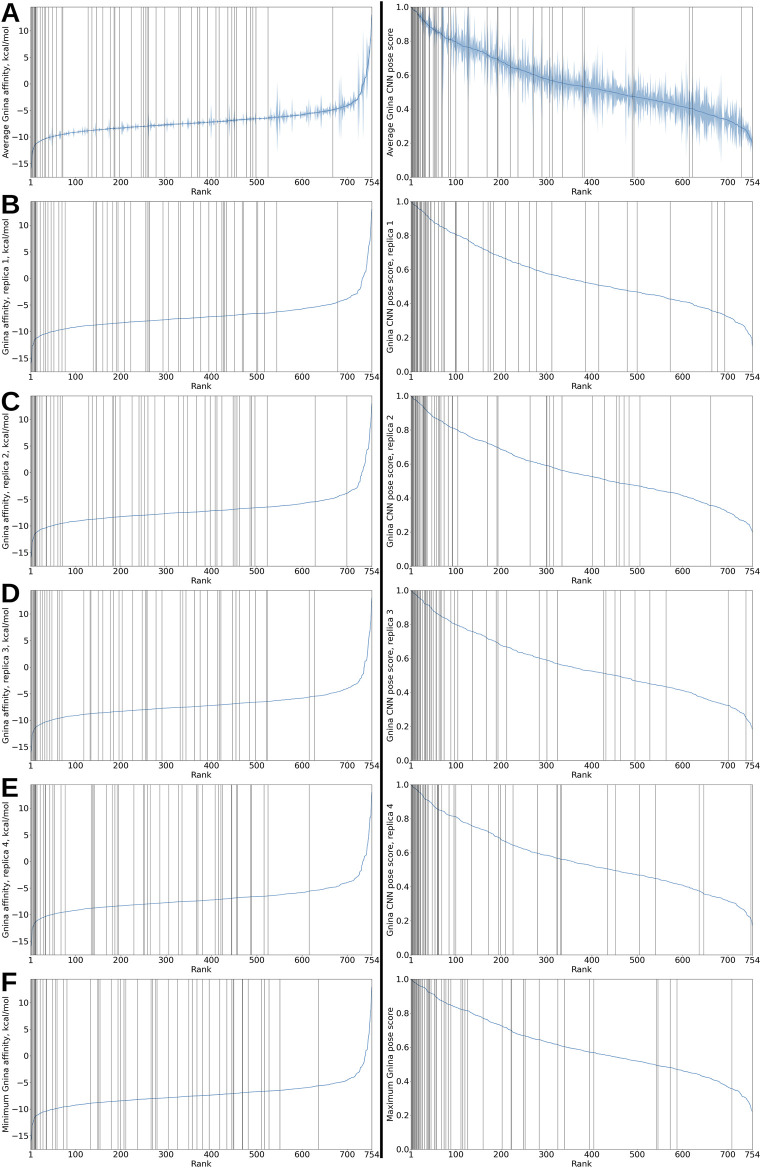
Recovery plots for Gnina affinity and CNN pose score on the PDBbind-derived set. In all panels, the vertical black bars indicate binding protein–ligand pairs. **(A)** Left. Protein–ligand pairs sorted by mean Gnina affinity, averaged over the 4 docking runs. The blue shaded region represents the score intervals at 95% confidence corresponding to every rank. Right. Protein–ligand pairs sorted by mean Gnina CNN pose score, averaged over the 4 docking runs. The blue shaded region represents the score intervals at 95% confidence corresponding to every rank. **(B)** Left. Protein–ligand pairs sorted by Gnina affinity from replica 1. Right. Protein–ligand pairs sorted by Gnina CNN pose score from replica 1. **(C)** Left. Protein–ligand pairs sorted by Gnina affinity from replica 2. Right. Protein–ligand pairs sorted by Gnina CNN pose score from replica 2. **(D)** Left. Protein–ligand pairs sorted by Gnina affinity from replica 3. Right. Protein–ligand pairs sorted by Gnina CNN pose score from replica 3. **(E)** Left. Protein–ligand pairs sorted by Gnina affinity from replica 4. Right. Protein–ligand pairs sorted by Gnina CNN pose score from replica 4. **(F)** Left. Protein–ligand pairs sorted by minimum Gnina affinity from the four replicas. Right. Protein–ligand pairs sorted by maximum Gnina CNN pose score from the four replicas.

**FIGURE 2:**
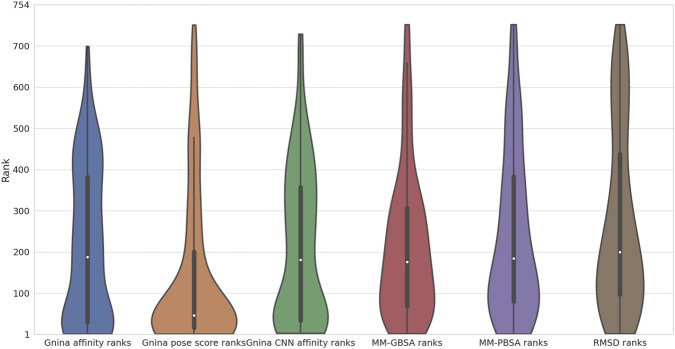
Ranking violin plots. The binding protein–ligand pair ranks from the four replicas when sorting by Gnina affinity, CNN pose score, CNN affinity, MM-GBSA, MM-PBSA, and RMSD are given. The RMSD is the ligand heavy atom RMSD at the end of production dynamics compared to at the end of minimization. The white dots represent the medians of the distributions; the thick black bars are the interquartile ranges; the thin black bars are the remaining data points.

We begin by examining the rankings produced by docking and molecular dynamics on the PDBbind-derived set. It contains a total of 754 protein–ligand pairs, 55 of which are binding (29 redockings and 26 crossdockings) and 699 nonbinding for an active:inactive ratio of around 1:12. Scoring all 754 pairs and ordering them by score produces a monotonic score vs. rank function; marking the positions of the actives produces a plot henceforth referred to as a *recovery* plot. In an ordered list of scores, a perfect estimator would place all 55 binding pairs at the beginning of the list; a perfectly random predictor would space out actives evenly at a ratio of 1 active for every 12 or so decoys. Real scoring tools are assessed by their recovery capabilities–the more actives they place near the beginning of the list, the greater their recovery capacity and the greater their utility in a drug discovery setting. We first compare the rankings afforded by the Gnina affinity and CNN pose score from the four docking runs and compare them to each other as well as to the rankings produced by averaging across the four runs, as well as taking the extremum for every ligand from the four runs (Figure 1). Rather than being evenly spread throughout the ranking list, i.e., the X axis, the actives are predominantly concentrated near its beginning; this is more pronounced with the CNN pose score ranking. We also see that the rankings from the four replicas generally do not differ drastically; averaging scores across replicas or taking the best score for every ligand also do not appear to greatly enhance recovery. Crucially, both affinity and CNN pose score metrics rank many binding pairs well and place them near the beginning of the list. For this set, CNN pose score outperforms affinity in terms of recovery in that affinity places more actives in the middle or near the end of the list. The differences in recovery capacity become more apparent if we look at a violin plot of the ranks the different metrics assign to the actives in the 4 replicas (Figure 2).

The violin plot clearly demonstrates that for the PDBbind-derived set, of the three Gnina descriptors, CNN pose score achieves the best recovery. Of the three parameters, its distribution is most concentrated near 1 (the beginning of the list) – it has the lowest lying median (the white dots in the violin plots) and its interquartile range (the thick black bars in the plots) is the smallest and lowest-lying, i.e., it lies closest to 1. Conversely, this parameter has the lowest fraction of poorly ranked actives - we see that using CNN pose score, a small fraction of active pairs rank beyond 100. This is not the case with affinity and CNN affinity, which have similar distributions. Surprisingly, CNN pose score achieves superior recovery than either MM-GBSA or MM-PBSA which do not differ significantly in rank distributions.

### MAYGEN-derived set

**FIGURE 3:**
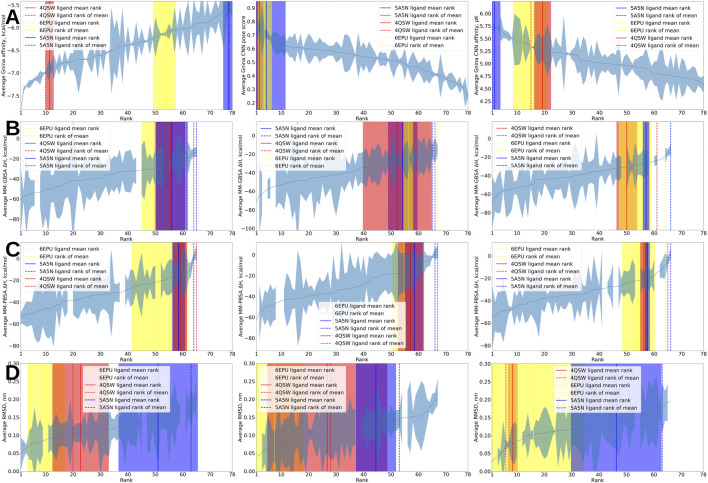
Recovery plots for Gnina affinity, CNN pose score, CNN affinity, MM-GBSA, MM-PBSA, and RMSD on the MAYGEN-derived set. In all panels, the vertical colored bars indicate binding ligands. The solid lines indicate the means of the 4 ranks, averaged across the 4 replicas; the dashed lines indicate the ranks of the mean scores, averaged across the 4 replicas. The blue shaded region around the curves represents the score interval at 95% confidence corresponding to every rank; the vertical shaded regions around the solid vertical lines represent the 95% confidence intervals for the corresponding ranks. The red shaded regions, centered on the solid red lines, represent the 95% confidence intervals for the ranks of the 4QSW ligand; the yellow and blue regions are for the 6EPU and 5A5N ligands, respectively. Note that the shaded regions often overlap. **(A)** Left. Ligands sorted by mean Gnina affinity, averaged over the 4 docking runs. Middle. Ligands sorted by mean Gnina CNN pose score, averaged over the 4 docking runs. Right. Ligands sorted by mean Gnina CNN affinity, averaged over the 4 docking runs. **(B)** Left. Ligands sorted by mean MM-GBSA values, averaged over the 4 MD runs. The simulations were started from the best scoring poses by Gnina affinity. Middle. Ligands sorted by mean MM-GBSA values, averaged over the 4 MD runs. The simulations were started from the best scoring poses by Gnina CNN pose score. Right. Ligands sorted by mean MM-GBSA values, averaged over the 4 MD runs. The simulations were started from the best scoring poses by Gnina CNN affinity. **(C)** Left. Ligands sorted by mean MM-PBSA values, averaged over the 4 MD runs. The simulations were started from the best scoring poses by Gnina affinity. Middle. Ligands sorted by mean MM-PBSA values, averaged over the 4 MD runs. The simulations were started from the best scoring poses by Gnina CNN pose score. Right. Ligands sorted by mean MM-PBSA values, averaged over the 4 MD runs. The simulations were started from the best scoring poses by Gnina CNN affinity. **(D)** Left. Ligands sorted by mean RMSD values, averaged over the 4 MD runs. The RMSD is the ligand heavy atom RMSD at the end of production dynamics compared to at the end of minimization. The simulations were started from the best scoring poses by Gnina affinity. Middle. Ligands sorted by mean RMSD values, averaged over the 4 MD runs. The simulations were started from the best scoring poses by Gnina CNN pose score. Right. Ligands sorted by mean RMSD values, averaged over the 4 MD runs. The simulations were started from the best scoring poses by Gnina CNN affinity.

For the MAYGEN-derived set, we keep track of the individual actives which allows us to estimate how much their rankings vary across the 4 replicas. Figure 3 shows the recovery plots for the averaged Gnina descriptors and MM-GBSA and MM-PBSA scores with the 95% confidence intervals for the ranks and scores. It shows that the ligand from the 4QSW structure can be expected to be placed by Gnina affinity between ranks 10 and 12 out of 78 total compounds (3 actives and 75 decoys) with 95% confidence, as estimated from the four docking runs. The 6EPU ligand can be expected to rank somewhere between 50 and 60; 5A5N consistently ranks poorly near the end of the list. Further, we obtain estimates for the variation in score at the corresponding ranks. For example, we see that for a compound to rank near the 50th spot by Gnina affinity, it needs to have a score between around −6.6 and −5.8 kcal/mol; the variation in scores increases near the end of the ranking. Conversely, there is much less variation in score at the beginning up to rank 10.

For the set we have here, with all three Gnina descriptors, there is a high degree of consistency between the means of the ranks from the 4 replicas (solid vertical lines in Figure 3) and the ranking produced by averaging scores and ordering the mean values (dashed vertical lines in Figure 3). Crucially, with the MAYGEN set, as well as with the PDBbind-derived set, CNN pose score is the top performer not only among the Gnina descriptors but overall–it ranks the three actives near the beginning of the ranking, unlike all other parameters which place one or all of the actives near the middle or the end. Although not all MD simulations succeeded and hence we do not have MM-GBSA and MM-PBSA scores for all ligands, it is evident the end-state techniques underperform–they clearly achieve inferior recovery to Gnina pose score, even on a smaller set of ligands. Moreover, MD-based scores show considerably more variation in score and ranking among the 4 replicas.

## Discussion

First, consider the active structures from Supplementary Figure S1. Then consider their respective isomers. Looking at the 2D structures, one can have a high degree of confidence that not even one of the isomers is an active molecule or a false negative. Similarly, for the PDBbind randomizations, one can be highly confident that the overwhelming majority of the randomized protein–ligand pairs do not bind each other with any appreciable affinity. Instead of synthesizing and experimentally testing every protein–ligand pair, the majority are discarded after an *in silico* evaluation, with experimental testing reserved only for the most promising compounds. These are some of the cost savings that justify and enable computer-aided molecular discovery and design. Remarkably, it is the high degree of selectivity and low random binding rates that enable modern pharmacotherapy (Gim and Vincent, 2013) in the first place. Indeed, were random binding rates much higher than 1% on average, it would likely be very difficult to administer a drug for a specific intended target without it perturbing some other important protein or pathway given that the human genome encodes around 20,000 proteins (Amaral et al., 2023) with additional splice variants (Wu et al., 2022) and macromolecules such as RNAs, DNA, and different macromolecular assemblies. To conclude this line of reasoning, we note that the primary utility of validation sets is in comparison–two or more pipelines are compared in terms of performance on the same data set or two or more modifications or variations of the same pipeline are tested. Recovery is evaluated using the known actives. Whatever false negatives may be present, be it due to alternative conformations of the primary binding site or any alternative binding sites, are treated equally as negatives until sufficient data become available to reclassify them as binders.

**FIGURE 4:**
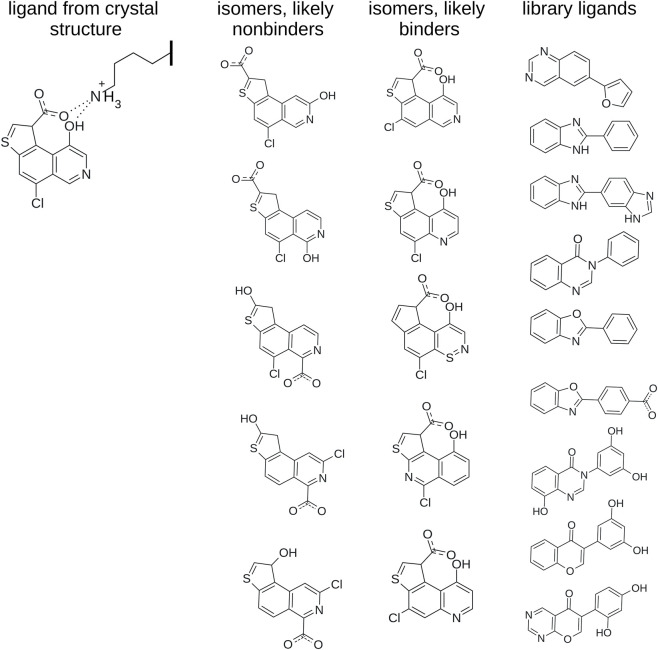
A hypothetical case of an experimentally validated pharmacophore observed in a crystal structure. The pharmacophore consists of a carboxylic and hydroxylic group simultaneously interacting with a lysine side chain from a protein. Separating these groups or placing them in locations where they cannot interact with the lysine side chain is likely to produce isomers that are inactive. Conversely, making small changes to the active ligand such as slightly altering the position of the chlorine or a heteroatom is likely to produce active isomers as long as the key pharmacophore remains intact. Typical biaryls from ligand libraries are also given for comparison.

Generating isomers is particularly easy and useful when there is a clear, well-established and experimentally validated pharmacophore that is known to be essential for ligand activity. This scenario is illustrated in Figure 4 where we have a hypothetical pharmacophore consisting of a carboxylic and hydroxyl group simultaneously interacting with a lysine side chain. In such a scenario, it would be easy to generate nonactive isomers by placing the hydroxyl group and carboxylate far apart and away from the lysine side chain. Such ligands would be far more suitable comparisons to the active ligand than the typical biaryls which are often found in drugs and druglike molecules (Dahlgren et al., 2013) and ligand libraries (Hussain et al., 2016; Boger et al., 1999; Stammers et al., 2023; Clark et al., 2023). Notably, in such cases, isomerization can likely generate active ligands as well. Given the active molecule in Figure 4, for example, shifting the position of the chlorine slightly or moving around one of the heteroatoms is not likely to abrogate activity, as long as the key pharmacophore remains intact.

Here, we present a rigorous means of estimating recovery and enrichment at every step of a VHTS pipeline in order to lay out a blueprint for future validation studies. The actives:inactives ratio in our PDBbind set is around 1:12 (55 binding pairs to 699 nonbinding pairs), with the actives spread nearly equally between redocking and crossdocking cases; the active to inactive ratio in our MAYGEN set is 1:25. We heavily emphasize that the tools and data set we use here are merely examples. A vast number of alternative techniques and combinations thereof are possible. We cannot conclusively say which if any one of them is definitively superior to the rest and, if so, what that is. We can only provide a method to tackle these questions which is a large undertaking for the entire computational chemistry community. Our set is small due to limited computational resources; it only serves to illustrate the principle of evaluating performance for every step and variance in rankings. In a prospective search, particularly in an industry setting, much larger sets with many more actives and decoys would have to be employed with dilutions up to 1:100 or better still 1:1000. Given access to cloud computing, such sets would be trivial to process (Lawrenz et al., 2015; Hande and Tamboli, 2024). Moreover, data sets would often have to be specifically prepared for the target at hand. For example, when targeting the pore of a GPCR ion channel, practitioners should assemble a large and diverse set of GPCR structures and binders. In such a scenario, globular proteins such as proteases or globins would be of little to no value and *vice versa*. Moreover, when targeting ion channels, practitioners would be advised to treat closed and open structures separately. Here, we can only provide general advice; we cannot hope to cover every eventuality. Ample positive data is available in PDBbind and in the literature. From then on, it is trivial to generate practically unlimited amounts of random protein–ligand pairs and isomers. Ivanov (2026) showcases code to generate and process isomers on a large scale with MAYGEN. It demonstrates that isomerization can be an efficient tool to generate decoys that match the actives more closely than the experimental decoys in key molecular properties such as molecular weight, charge, surface, numbers of hydrogen bond donors, acceptors, and rotatable bonds. Crucially, it shows that isomerization can produce decoys that are better positioned than the experimental decoys in property hyperspace, i.e., closer to the actives (Ivanov, 2026).

We present a means of thoroughly and rigorously assessing the value added by every step in a VHTS pipeline and showcase how this matter should be investigated. Any pipeline should be tested and validated in a similar manner and every step in it should be modified until it reliably and consistently improves enrichment and recovery, otherwise it should be removed. Methods which underperform should be swapped out in favor of ones which improve recovery compared to the previous step, which does not appear to be the case with Gnina and MM-GB(PB)SA in this instance. This is noteworthy because MM-GB(PB)SA results (Maraf et al., 2024; Poli et al., 2020; Kim et al., 2024; Sahakyan, 2021; Yazdani et al., 2023; Alturki et al., 2022; Ahmed et al., 2023; Okimoto et al., 2009) or even cruder metrics like atomic RMSDs or RMSFs (root mean square fluctuations (Menchon et al., 2018)) are employed as mid-to-late-stage filters in virtual high-throughput screens. Clearly, such pipelines should be validated and recalibrated using the approach we propose here. For the PDBbind set, for example, ranking ligands by RMSD is little different than ranking by MM-GB(PB)SA; for the MAYGEN set, ranking by RMSD arguably outperforms MM-GB(PB)SA, but exhibits a lot more variability from replica to replica. Here, MM-GBSA achieves recovery comparable to MM-PBSA, despite being a lower-level-of-theory technique, involving more simplifications, and costing about an order of magnitude less in calculations. This is consistent with our previous results on RNase A–ligand binding (Ivanov et al., 2019a), where MM-GBSA performed similarly to MM-PBSA on calculating relative ligand affinities. We once again stress that the tools we use here–MAYGEN, Gnina, and MM-GB(PB)SA–only serve as a story-telling device; they are mere illustrations of a more important principle. It is the *principle* that must be learned not the *example*. Use of the tools does not imply endorsement or recommendation, neither for, nor against. Indeed, the search for the optimal VHTS protocol very much remains ongoing.

While one can choose to omit either or both the isomerizations and randomizations during VHTS pipeline validation and use only set(s) one is interested in, we strongly encourage including both an isomerization and randomization set in the validation. By virtue of preserving heavy atom composition, isomerization sets are likely to be much less biased than sets of completely unrelated actives and inactives, although discrepancies and imperfections cannot be avoided completely. Thus, this is an astute means of *minimizing* bias. From this vantage point, we highlight two reasons for including the PDBbind randomizations in our analysis. First, results from the isomerization set (which is known to have ligand bias) must be compared to results from the PDBbind randomizations which do not have any ligand bias (because the same set of molecules serve as actives as well as inactives). Thus, if results are consistent between these two very different sets and whatever proprietary or publicly available set is being used, one has good reason to believe that what is being revealed is the true recovery capacity of the pipeline rather than some random outcome or the result of a bias in the isomerization or proprietary/open source set. Second, the protein–ligand randomizations constitute the ultimate benchmark for ABFE calculations which are believed to be approaching maturity. If this is truly the case, any sufficiently mature and capable ABFE pipeline (Ries et al., 2024; Heinzelmann and Gilson, 2021; Heinzelmann et al., 2024) should consistently outperform docking and end-state free energy techniques which involve multiple simplifications and approximations. Pairing ligands to different proteins and correctly ranking their affinities is a harder challenge and a more stringent test than ranking ligands with the same protein. For example, a benzodiazepine simulated with the steroid receptor or a steroid molecule simulated with the benzodiazepine receptor should produce less favorable ΔG_calculated_ values than the cognate binding pairs. By their very nature, ABFE calculations are the only technique which should be expected to consistently succeed at this more stringent test. However, no such tests have thus far been carried out and it is presently unclear if current ABFE pipelines would pass even this conceptually simple check.

With deterministic sampling tools, e.g., OpenEye’s OMEGA (Hawkins et al., 2010) and FRED (McGann, 2011), running the same task with the same settings multiple times produces identical results (within machine precision), so there is no added benefit, only added cost, to performing the same task more than once. This is not the case with stochastic sampling tools and algorithms, such as Gnina or Molsoft’s ICM (Abagyan et al., 1994), which randomly sample possible ligand conformations and which might uncover new, more favorable, and previously unsampled conformations, given more runtime. It is then worth considering where molecular dynamics lies on the spectrum between fully deterministic and stochastic sampling techniques. In the hypothetical case of perfect sampling, where the entirety of available conformational space has been explored, MD results would be practically identical regardless of the starting conformation or velocities. Such simulations are said to be ergodic (Karplus and McCammon, 2002). In practice, however, MD runs often sample only parts of the available conformational space, with different runs reaching different subregions with varying degrees of overlap between the conformations sampled in different runs. This means that MD simulations are often quasiergodic rather than ergodic (Chipot, 2023). This makes it necessary to estimate the variability in results and rankings one can expect to obtain with stochastic (e.g., Gnina or ICM docking) or quasiergodic techniques (e.g., MM-GBSA or ABFE calculations). We demonstrate how this variability can be estimated. Our work on the MAYGEN set demonstrates that with MM-GBSA or MM-PBSA, rankings can vary up to 10 positions, potentially even more, which is a significant margin given that the set contains 78 molecules in total. Moreover, we see that RMSD is too crude of a metric–the rankings it produces can vary up to a third, potentially even half, of the span of the X axis. This is too wide a margin for a ranking to be considered reliable. Further, it is important to estimate how the variance in ranking scales with increasing dilution, as the actives:decoys ratio reaches and surpasses 1:50, 1:100, 1:1000, and beyond, reaching levels found in real-world screening decks. We leave this matter as the subject of future reports, by ourselves and hopefully others as well.

We also stress that the method we lay out here should be used to first calibrate VHTS pipelines on the target of interest, i.e., to asses the baseline enrichment and recovery metrics at every step given the specific target and ligand deck at hand. Among practitioners, it is known that different classes of proteins can vary greatly in terms of difficulty for hit discovery - kinases tend to be relatively easy (by the standards of hit discovery), proteases are usually more difficult, metaloenzymes are typically very difficult. Indeed, hit rates can vary by up to several orders of magnitude from target to target and from ligand deck to ligand deck. Thus, one should not expect good hit rates from one target to immediately translate to another. Rather, a baseline should be established for every class of target, ideally for every protein target at hand. Then, this baseline should be used to validate any modifications to the pipeline. Hydrogen mass repartitioning, for example, is often employed to speed up MD simulations (Hopkins et al., 2015). Whether or not it affects recovery, in what way, and in what context, however, have thus far not been explored. Other avenues which warrant further exploration are the influence of adding ligand solvation energies (Ivanov, 2024a) to pipelines which do not have them, ligand strain energies (Tong and Zhao, 2021) and rototranslational entropies (Irudayam and Henchman, 2009; Chang et al., 2007) to name a few, many more can be envisaged and tested. Ranking and recovery analysis, as presented here, can also be used in force field development (and method development more broadly) as a test that is orthogonal (and hence independent) from the metrics used to tune and validate force fields or water models such as enthalpies of melting or evaporation (Warshel and Lifson, 1970), melting points of water (Xiong et al., 2020), charge distributions (Izadi et al., 2014), radial distribution functions (RDFs, usually the oxygen–oxygen RDF for water models (Izadi and Onufriev, 2016)), QM-derived energies and parameters (Tian et al., 2020), etc. Results from both the PDBBind and MAYGEN sets clearly demonstrate that the Gnina CNN pose score metric achieves superior recovery to Gnina affinity and Gnina CNN affinity. This is the exact same outcome that was observed during Gnina’s development (McNutt et al., 2021), despite the fact that the Gnina developers used different proteins, ligands, and protein–ligand pairs from the ones used here. In fact, the Gnina developers did not even have access to the MAYGEN decoys used herein. This work, therefore, corroborates and validates the developers’ decision to make pose score the default selection criterion using a test that is very different from the one used during Gnina’s development (McNutt et al., 2021). More generally, it illustrates that testing and validation should be done using at least two orthogonal sets, and shows that robust and reliable conclusions can only be drawn after arriving at the same outcome from very different starting points.

Supplementary Figure S2 shows a breakdown of the rankings afforded by some of the terms that go into the final MM-GBSA and MM-PBSA results and compares them to the final MM-GBSA and MM-PBSA rankings. The figure lists the rankings by polar solvation energy (EGB), nonpolar solvation energy (ESURF), total gas phase (G GAS), and total solvation energy (G SOLV) from the MM-GBSA calculations. For MM-PBSA, it lists polar solvation (EPB), nonpolar solvation (ENPOLAR), total solvation (G SOLV), as well as the dispersion terms (EDISPER) that go into the ENPOLAR terms in MM-PBSA, as well as the gas phase energies (G GAS) which are the same for MM-GBSA and MM-PBSA. We see that the combination of the terms that produces the final MM-GBSA and MM-PBSA results and rankings has superior recovery to the individual terms, thereby offering an orthogonal verification of the end-state free energy techniques and their underlying theory. It has also not evaded our notice that some of the least theoretically rigorous terms in MM-GB(PB)SA–the solvation terms and the polar solvation terms in particular–have some of the lowest recovery capacities. Furthering the previous line of reasoning, we conjencture that using more rigorous solvation schemes (Ivanov, 2024a) and adding a term for entropy (potentially more than one, e.g., ligand configurational entropy (Chang et al., 2007), side-chain conformational entropy (Doig and Sternberg, 1995), rototranslational entropy (Fogolari et al., 2018), etc.) that is (are) of sufficient quality and theoretical rigor should further improve recovery to the point where it consistently outperforms docking, potentially becoming competitive with alchemical ABFEs (Lee et al., 2020; Macchiagodena et al., 2020; Cournia et al., 2020; Aldeghi et al., 2016).

We caution the novice practitioner to carefully consider the output from VHTS tools and pipelines and not overrely on and overtrust their output. As an example, note that the majority of protein–ligand pairs in both the PDBbind- and MAYGEN-derived sets have a predicted Gnina affinity below −6 kcal/mol (see the left panels in Figure 1 and the left panel in Figure 3A). Despite the fact that the results are given in kcal/mol, these should not be viewed as absolute binding free energies (which would correspond to dissociation constants below 40 μM which is appreciable binding) but relative to each other at best. Indeed, given the inherently low positive rates for random protein–ligand pairs, it is likely that not even one protein–ligand pair other than our designated positives have any appreciable affinity for each other.

With the present report, we hope to focus the attention of the research community on the need for sufficient and high quality negative data. Again, we do not view our current data sets as definitive or conclusive; we are merely laying out the blueprint for larger studies with positive:negative ratios exceeding 1:100, hopefully 1:1000. As a further means of making the validation more stringent and rigorous, protein structures could be replaced by alternative ones (Hahn et al., 2022) such that only crossdocking is performed. It is well established that redocking is an easier task than crossdocking (Röhrig et al., 2023). This is also evident from our own work, where ranking by any parameter produces superior recovery in cases of redocking versus crossdocking (Supplementary Figure S3). To make the test even harder and the validation still more stringent, the pipeline could be initiated using apo- rather than holostructures (Zhang et al., 2023).

Furthermore, high quality negative data is needed not only to accurately estimate the capabilities of VHTS pipelines in a hit discovery setting; it is also necessary to fairly estimate the capabilities of relative binding free energy (RBFE) methods in hit-to-lead progression and lead optimization. Strikingly, examples of machine learning algorithms trained exclusively on positive data have been published (Jiménez et al., 2018). This is completely unrealistic in drug discovery where the supermajority of ligands are decoys, i.e., do not actually bind the target. If all ligands an ML model or artificial intelligence (AI (Blaschke et al., 2020; Guo et al., 2021; Arús-Pous et al., 2020; Fialková et al., 2021; Guo et al., 2023; Sundin et al., 2022; Moore et al., 2022; Wang et al., 2022)) encounters during training are binders, this reinforces the utterly incorrect implicit supposition that every ligand the model encounters is a binder, even if only a weak one (Leckband and Sivasankar, 1999). Similarly, instances of RBFE or even ABFE studies conducted on data sets that do not have any negative data points have been published (Aldeghi et al., 2016; Moore et al., 2023; Ross et al., 2023; Aldeghi et al., 2017). Worse still, in many cases most of the ΔG_bind_ values are much lower than 0 kcal/mol (ΔG_bind_ << 0 kcal/mol) or are very few in number and span a narrow affinity range (Fratev et al., 2018). Studies like these do not assess the tools’ ability to identify nonbinding congeners. We, therefore, recommend that negative data of sufficient quality and quantity in the form on nonbinding congeners be added to the best practices requirements for carrying out protein–ligand RBFE benchmarks (Hahn et al., 2022) and commend reports that have data points with ΔG_bind_ below 0 but close to 0, as well as data points with ΔG_bind_ > 0 kcal/mol (Wade et al., 2022; Zhang et al., 2021). We emphasize that for RBFE studies, nonbinding *congeners* should be used as negative data not random isomers.

Our work demonstrates that despite the boastful reports of hit discovery using VHTS that abound in industry and academia alike, VHTS hit discovery is still very much reliant on the underlying random hit rate for the screening deck at hand. At best, VHTS pipelines only provide enrichment on top of that. Note, for example, that in the MAYGEN set, many of the industry-wide tools and techniques fail to place even 1 active in the top 10% (which is 8 ligands in this case). Indeed, out of a set of 78 ligands with 3 actives, only Gnina CNN pose score consistently ranks two of the actives in the top 10% (see Figure 3). Almost all other metrics–Gnina affinity, MM-GBSA, MM-PBSA, and ligand RMSD–would likely fail to place even one active in the top 10% even in such a small set where the ratio of actives to inactives is several orders of magnitude greater than what is likely to be found in real screening decks. While it could be argued that there are many more tools, techniques, and modifications to the workflow that could be attempted, these results certainly warrant a healthy amount of caution and skepticism. Given that some of the most common tools and approaches do not place even 1 active in the top 1% out of 75 decoys that are utterly structurally different from the actives, how likely is it that another tool or method would be able to find the same active among 1 million or 1 billion decoys, some of which will be very similar to the active, some will be very different, with many more anywhere in between? And even if the active is within the top 1% out of millions to billions of decoys, it could still be buried under thousands to millions, potentially tens of millions of decoys. Clearly, finding 1 active out of 1M+ decoys is a very tall order, and what hits VHTS is currently furnishing are likely thanks to the random hit rate (probably within the 0.1%–1% range in most cases), with whatever enrichment it provides added on top of that. Accordingly, in the CACHE challenges, hit rates in a truly prospective setting have thus far tended to be between 0% and 1% (Herasymenko et al., 2025) with teams rarely exceeding success rates of 5% (Gutkin et al., 2024; Mettu et al., 2025; Eguida et al., 2024). Notably, in the first CACHE challenge, a docking-based search (Dunn et al., 2024) was tied for first place with pipelines based on much more sophisticated algorithms involving molecular dynamics, ABFEs, machine learning or deep learning. This is a clear indication that these methods and algorithms often do not work as well as their developers and users would hope and that much testing and validation remain ahead.

We also note that in its current implementation, MAYGEN produces a lot of unphysical structures that are allowed by sheer element valences but disallowed by other constraints. For example, it tends to produce a lot of structures with nonlinear consecutive double bonds, especially in rings, which are unrealistic. This creates the need to manually filter out such structures. We are hopeful that the MAYGEN development team will address these shortcomings and further enhance its utility and practicality.

Lastly, we reiterate that any criticism levied herein is so levied with only positivism and constructivism as its ultimate end. Herein, we build upon the ideas, tools, and work of others in a collaborative effort to make a meaningful improvement and move the field toward maturity and delivering the new medicines so very much needed in the 21st century.

## Data Availability

The original contributions presented in the study are included in the article/Supplementary Material, further inquiries can be directed to the corresponding author.
