# Supplementary material for: An efficient computational chemistry approach to generating negative data for drug discovery pipeline validation
Source: Front Bioinform. 2026 Feb 27;6:1756279. doi: 10.3389/fbinf.2026.1756279 (PMC12982188; doi:10.3389/fbinf.2026.1756279)
Supplement: Supplementary file 1 [file Supplementaryfile1.docx]

**Table 1.** PDBbind sheet. The 754 protein – ligand pairs and their binding status (binding/nonbinding) are given. The first pair is the 1FCX ligand docked to the protein from the 1FCX crystal structure (redocking), the second is the 1G74 ligand docked to the protein from 1FCX (crossdocking), the third is the 2P3I ligand docked to the 1FCX protein (crossdocking), etc. Prot_XXXX is the protein structure (taken from PDB XXXX), lig_YYYY is the ligand structure (taken from PDB YYYY). Generally, when XXXX = YYYY, the pair is a binding one, when XXXX != YYYY, the pair is a nonbinding one except when PDB structures XXXX and YYYY contain the same protein. MAYGEN Sheet. The 4QSW, 5A5N, 6EPU ligands, and their structural isomers used in the present study, are given in SMILES format.


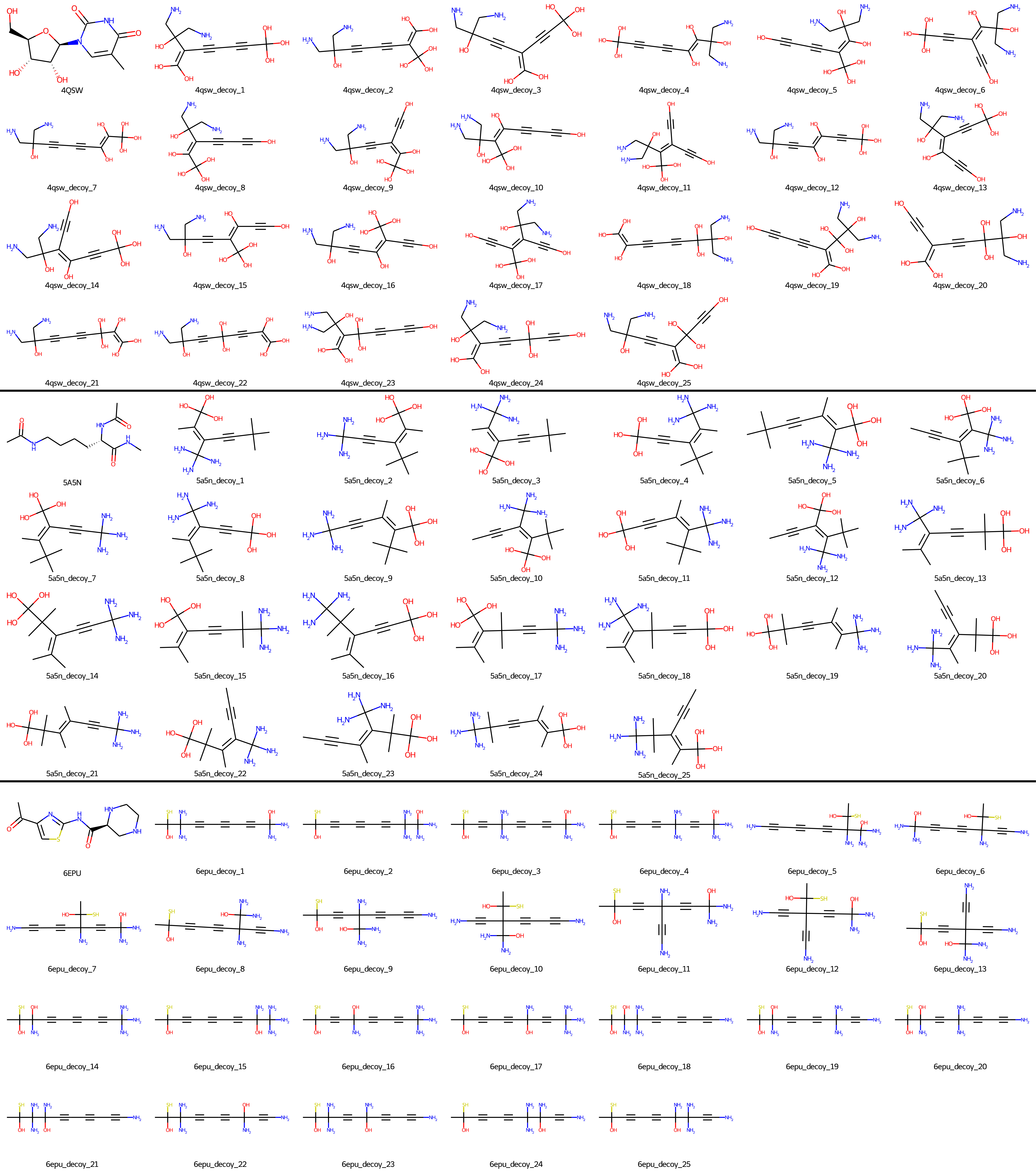


**Figure S1. MAYGEN set ligands.** The structure of the 4QSW ligand is given, followed by 25 of its structural isomers used in the present study, followed by the 5A5N ligand and its isomers, followed by the 6EPU ligand and its isomers.


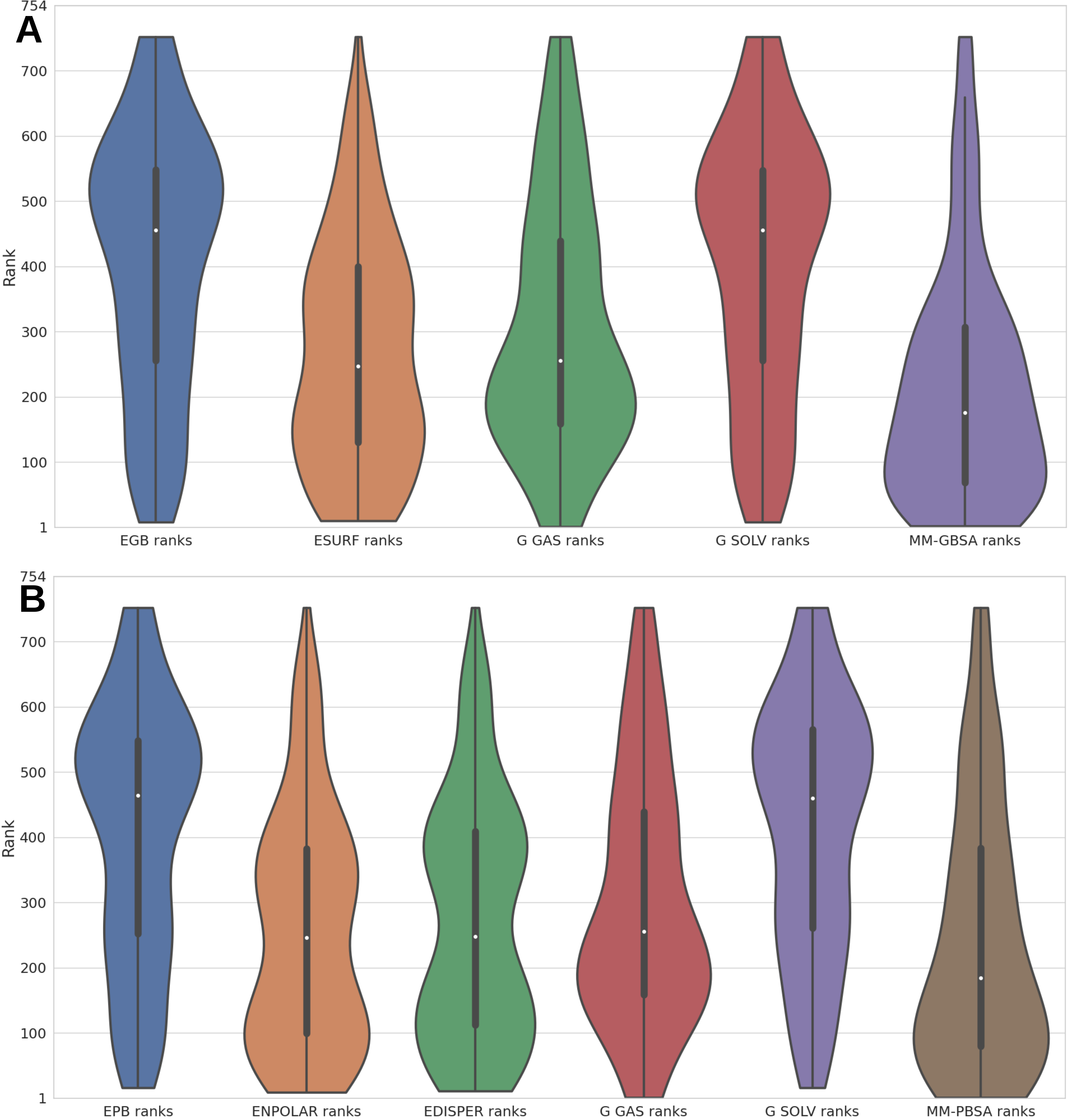


**Figure S2. Ranking violin plots.** (A) The binding protein – ligand pair ranks for the four replicas when sorting by polar solvation energy (EGB), nonpolar solvation energy (ESURF), total gas phase (G GAS), total solvation energy (G SOLV), and total MM-GBSA energies. (B) The binding protein – ligand pair ranks for the four replicas when sorting by polar solvation energy (EPB), nonpolar solvation (ENPOLAR), dispersion energy (EDISPER), total gas phase (G GAS), total solvation (G SOLV), and total MM-PBSA energies. The G GAS terms go into both the final MM-GBSA and MM-PBSA results and are identical for MM-GBSA and MM-PBSA. The white dots represent the medians of the distributions; the thick black bars are the interquartile ranges; the thin black bars are the remaining data points.


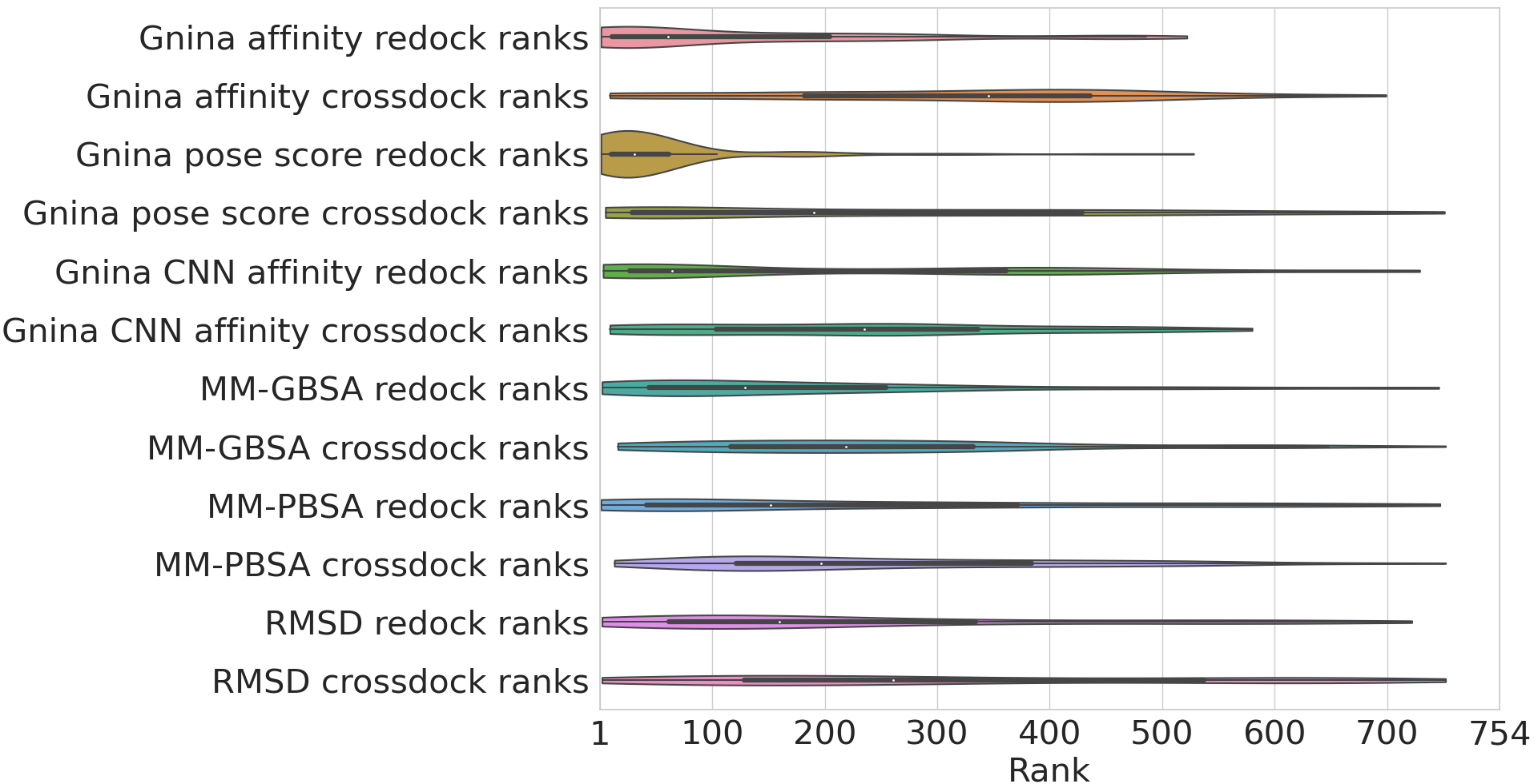


**Figure S3. Ranking violin plots.** The binding protein – ligand pair ranks for the four replicas when sorting by Gnina affinity broken down by redocking and crossdocking, followed by Gnina pose score redocking and crossdocking ranks, followed by Gnina CNN affinity ranks, MM-GBSA, MM-PBSA, and RMSD ranks. The RMSD is the ligand heavy atom RMSD at the end of production dynamics compared to at the end of minimization.
